# Cell-specific metabolomic responses to injury: novel insights into blood-brain barrier modulation

**DOI:** 10.1038/s41598-020-64722-w

**Published:** 2020-05-08

**Authors:** Sheng-Fu Huang, Sabrina Fischer, Alexey Koshkin, Endre Laczko, David Fischer, Omolara O. Ogunshola

**Affiliations:** 1 Institute for Veterinary Physiology, University of Zurich. Winterthurerstrasse 260, CH-8057 Zurich, Switzerland; 2 Zurich Center for Integrative Human Physiology, University of Zurich. Winterthurerstrasse 190, CH-8057 Zurich, Switzerland; 3 Institute of Zoology, University of Basel, Vesalgasse 1, CH-4051 Basel, Switzerland; 4 Functional Genomics Center Zurich, University of Zurich, Winterthurerstrasse 190, CH-8057 Zurich, Switzerland

**Keywords:** Metabolomics, Extracellular signalling molecules

## Abstract

On one hand blood-brain barrier (BBB) disturbance aggravates disease progression, on the other it prevents drug access and impedes therapeutic efficacy. Effective ways to modulate barrier function and resolve these issues are sorely needed. Convinced that better understanding of cell-oriented BBB responses could provide valuable insight, and the fact that metabolic dysregulation is prominent in many vascular-related pathological processes associated with BBB disturbance, we hypothesized that differential cell-specific metabolic adaptation majorly influences physiological and pathological barrier functionality. Untargeted liquid chromatography–mass spectrometry (LC-MS) metabolomic profiling was used to obtain individual biochemical fingerprints of primary astrocytes (AC) and brain endothelial cells (EC) during normoxic conditions and increasing hypoxic/ischemic injury and thus a functional readout of cell status. Bioinformatic analyses showed each cell had a distinct metabolic signature. Corroborating their roles in BBB and CNS protection, AC showed an innate ability to dynamically alter their metabolome depending on the insult. Surprisingly, in complete contrast, EC largely maintained their normoxic characteristics in injury situations and their profiles diverged from those of non-brain origin. Tissue specificity/origin is clearly important when considering EC responses. Focusing on energy capacity and utilization we discuss how cell-specific metabolic adaptive capabilities could influence vascular stability and the possibility that altering metabolite levels may be an effective way to modulate brain EC function. Overall this work novel insight into cell-associated metabolic changes, and provides a powerful resource for understanding BBB changes during different injury scenarios.

## Introduction

Stability of the neurovascular unit (NVU) as a result of proper blood-brain barrier (BBB) function is essential for brain and whole body homeostasis. BBB dysfunction occurs in many brain pathologies and increased BBB permeability directly correlates with poor disease outcome^1–3^. In contrast the BBB also actively prevents drugs aimed at improving neuronal function from gaining access to the brain tissue. It seems clear that patient recovery after injury would benefit from controlled modulation of BBB permeability. Unfortunately this goal remains an unsolved challenge.

Located at the level of the impermeable brain microvasculature, the BBB maintains CNS homeostasis by regulating the passage of ions and molecules between the brain and circulatory system^1,3^. The brain endothelial cells (EC) that form the barrier are supported by perivascular cells, namely astrocytes (AC) and pericytes, that secrete growth factors and substrates to sustain proper barrier function^1,3^. We still have limited understanding of how these barrier-associated cells respond individually or interact concertedly - physiologically or pathologically. Particularly how changes in cell-specific metabolism relate to barrier homeostasis remains largely unclear. Better insight could offer more innate, and potentially highly effective, ways to modulate barrier characteristics.

To date studies on endothelial metabolism have focused on peripheral EC isolated from lung^4^, arteries^5^ and retina^6^ as well as human umbilical vein EC (HUVEC)^7^. Mitochondrial respiration in these non-brain EC is relatively low compared to other cell types. Most quiescent non-brain EC are primarily glycolytic, using β-fatty acid oxidation to generate ATP, maintain redox homeostasis as well as support nucleotide and protein synthesis^8^. This basal metabolic profile becomes even more glycolytic during injury with higher activity in glutamate and pentose phosphate pathways (PPP) enabling replenishment of critical energy stores to support hypoxic angiogenesis^9,10^. Similar mechanisms are also activated during cancer EC proliferation with glutamate and glucose being utilized to increase energy generation and nucleotide synthesis during hypoxia and ischemia^11^.

Detailed metabolomic profiling of brain microvascular ECs has not been published to date. The brain endothelium is unique having a distinct proteomic composition and exhibiting unexpected molecular alterations during injury such as elevated protein synthesis^12^, suggesting specialized processes continue despite challenging conditions. Furthermore, angiogenesis, the state that leads to increased vascular permeability, is activated mainly during prolonged or extreme injury conditions in brain EC^13,14^. These unique properties most likely augment their critical barrier role and suggest a detailed study is warranted to better understand their unique properties.

AC influence both BBB function and the neurovascular unit (NVU) as a whole. Since their endfeet contact both vessel walls and neurons, they are important mediators of signals to and from the different cellular compartments^1,15,16^. AC metabolism is tuned to constantly facilitate brain homeostasis. Highly activated energy production, via TCA and glutamate recycling, enables AC to deal with high metabolic stress^17^. AC also boost glycogen degradation to replenish energy stores^18,19^ and generate glutathione and NAD^+^ to maintain cellular redox balance^20,21^ during brain injury. This inherent ability to rapidly adapt metabolically is crucial for surrounding neurons^22^ but whether similar or distinct metabolic coordination occurs between AC and nearby capillaries is unknown.

We aimed to obtain detailed insight into individual AC and EC responses to environmental change by profiling BBB cell-specific metabolomic changes during different conditions. A number of challenges are standardly encountered when studying the BBB *in vivo* but in particular, it is problematic to understand where specific signals originate and/or the effects of changing conditions on individual cell populations. To circumvent these obstacles we performed metabolomic profiling of unpassaged primary AC and primary brain microvascular EC. Untargeted liquid chromatography–mass spectrometry (LC-MS) provided a global overview of cellular metabolite composition before and after exposure to increasing severities of hypoxia and/or ischemia simulating various pathological situations. By comparing baseline (normoxic) and injury-mediated changes in metabolite levels we show that AC and brain EC profiles differ significantly and that brain EC profiles also diverge from those of non-brain origin. Assessing key metabolic pathways involved in energy capacity and utilization, we conclude that stressed EC are likely to be highly reliant on metabolic support from surrounding cells. Indeed, the observed dynamic adaptive capacity of AC during insult presumably provides an important metabolic reservoir and underlies their ability to improve EC function and integrity. Overall, this study provides new insight into cell-specific metabolic changes, and provides a powerful resource for understanding BBB changes during different injury scenarios.

## Material and Methods

All animal experimental protocols in this study were approved by the University of Zurich Animal Protection Office and Swiss Veterinary Office of Canton Zurich, and conform to Swiss Animal Protection guidelines and regulations (Swiss Animal Protection and Swiss Animal Act and Ordinance).

### Primary cell isolation

All cell culture media and reagents were obtained from Gibco^®^ (Life Technologies, Zug, Switzerland) and Sigma-Aldrich (Buchs, Switzerland). Primary rat astrocytes (AC) were isolated from neonatal pups as described previously^23^, cultured in DMEM containing 5 mM glucose and supplemented with 10% FBS and 50 µg/ml gentamycin sulfate. Primary rat brain microvascular endothelial cells (EC) were isolated from 8–10 week old animals according to Coisne *et al*.^24^. Isolated EC were cultured in endothelial media (DMEM (5 mM glucose) supplemented with 20% newborn calf serum, 1x BME amino acids, 1x vitamin solution, 2 mM L-glutamine, 1 ng/ml bFGF, 50 µg/ml gentamycin sulfate) and plated on collagen-IV coated dishes. EC reached 100% confluency within 7 days and were used without passaging. Isolation purity was ≥95% as analyzed by immunostaining for standard cell markers (Fig. S1a). AC were identified by high GFAP expression and absence/low expression of NG2 and PDGFRβ (pericyte markers) and PECAM-1 (EC marker). Purity of EC monolayers was assessed by absence of GFAP, NG2, α-smooth muscle actin (smooth muscle cells) as well as localization of tight junction proteins (occludin, ZO-1 and Claudin-5) and PECAM-1 to cell-cell borders.).

### **O**_**2**_**deprivation and ischemic treatment**

O_2_ deprivation experiments were carried out in purpose-built hypoxic glove-box chambers (InVivO2 400, Ruskinn Technologies, Pencoed, UK) maintained at 37 °C with 5% CO_2_. O_2_ concentration was constantly monitored with an internal O_2_ sensor. To enable direct comparison of all results, both cell types were preincubated in MS (mass spectrometry) media (DMEM + 10% calf serum + BME amino acids, vitamin solution, 2 mM L-glutamine, 1 ng/ml bFGF and 50 μg/ml gentamycin sulfate) for 6 h. Fresh media was the applied to all plates immediately before exposure to normoxic (NX, 21% O_2_), hypoxic (HX, 1% O_2_) or near anoxic (AX, 0.2% O_2_) conditions for 24 h. Glucose-free media was used to simulate ischemia *in vitro* (oxygen-glucose deprivation (OGD).

### Specific quantitative Biochemical analysis

After exposure cell lysates were collected. ATP levels were detected using a fluorometric kit (Abcam, range = 0.1–1 nmol, sensitivity < 1 μM). Glucose levels were measured using a colorimetric kit (Abcam, range = 1–1000 μM, sensitivity = 1 μM). Cellular glycogen was detected using a fluorometric assay (Abcam, range = 0.4 µg/ml − 2000 µg/ml, sensitivity > 0.4 µg/ml). Samples were measured in duplicate and normalized to total cell counts obtained using DAPI staining and EVOS FL Auto Imaging System (Thermo Fisher Scientific). Four independent experiments were performed, N = 4.

### Metabolite extraction

After exposure to different conditions cells were washed twice with ice-cold 5 mM NH_4_HCO_3_ solution and residual liquid was removed. Cell metabolism was immediately quenched in 100 µl 80% methanol (−20 °C) containing 20 µg/ml C13/N15 labelled amino acid mixture (Cortecnet, Voisins-Le-Bretonneux, France) as the internal standard. Cells were scraped from the dish and incubated for 10 min on ice. After centrifugation (6000 g for 5 min at 4 °C) the supernatant containing the metabolites was lyophilized and stored at −80 °C until measurement. Four independent experiments were performed, N = 4.

### Untargeted LC-MS measurements

All mass spectrometry measurements and analyses were conducted in cooperation with the Functional Genomics Center Zurich (FGCZ), University of Zurich. Lyophilized metabolite pellets were resuspended in 20 µl ddH_2_O and transferred to 96-well plates. Samples were then diluted 1:5 in injection solution (90% acetonitrile, 10% methanol, 50 mM ammonium acetate, pH 9) and centrifuged. 30 µl of supernatant (duplicate) were transferred to a fresh 96-well plate and directly analyzed on a nanoACQUITY system coupled to a Synapt G2HD mass spectrometer (Waters Corp., Milford, USA). Chromatographic separation of metabolites was performed on a 0.2 µm × 150 mm BEH amide column using a 10 minute linear gradient of 90% to 50% acetonitrile, 0.5 mM ammonium acetate, pH 9. All analyses were done in negative mode using 1.2 kV capillary voltage, 30 V sampling cone voltage and 3 V extraction cone voltage. The source temperature was set to 100 °C and Nano Flow Gas, i.e. sheet gas flow, was applied. For quality control we spiked a mix of C^13^/N^15^ labelled amino acids during the extraction to follow the overall performance of the LC-MS method and data normalization. To estimate the overall quality of the quantification, we determined the RSD of two major metabolites for the reported HILIC methods, namely aspartic acid and glutamic acids. An RSD of 25.7% without total ion count normalization and 22.3% after normalization was measured for glutamic acid. For aspartic acid, RSD before normalization was 25.6% and 22.3% after normalization.

### Data analysis and processing

Waters raw data were first converted to centroid mode and further processed into vendor independent netCDF format using DataBridge (Masslynx, Waters Corp.). Untargeted metabolomics data matrix comprising of accurate mass/retention time information, and ion counts for each sample were calculated using the data processing tool cosmiq^25^. Signal-to-noise ratio (SNR) of mass peak detection was set to 3, SNR for chromatographic peak detection was set to 10 and a m/z bin size of 0.003 Da was chosen as parameters for cosmiq. For metabolite annotation, the results were first matched to a list of metabolites with known retention time and mass. For annotation of unknown metabolites, the list of accurate masses was matched to the KEGG database assuming [M-H]- adducts^26^. Database hits within a mass window of 0.01 Da were considered.

### Data normalization strategy

To compare the relative metabolite quantities between the different treatments and cell types, we performed a normalization approach according to the sum of all detected metabolite ion counts as previous^27^. This was important as hypoxia and ischemia differentially alter protein amounts, proliferation rates and DNA content. AC and EC also have significantly different cell size. The strategy avoids these confounding factors as it is based on the assumption that the total number of cells used for extraction is reflected by the total amount of metabolites and hence can be estimated by the sum of measured ion counts. One of the NX + Glc EC samples was randomly chosen as reference and the normalized ion intensity for each metabolite was calculated by dividing the observed ion intensity by the factor Σis/ Σir, where Σis is the summed ion intensity for each individual sample and Σir is the summed ion intensity of the reference sample.

### Pathway analyses

Statistically significant metabolites were analyzed using the KEGG metabolic pathway map^26^ and MetaboAnalyst^28^. The KEGG pathway map was retrieved in the KGML data format and imported into Cytoscape^29^ using the KGMLReader tool. Fold changes and significance values of the annotated metabolites were mapped on this global pathway map. A list of KEGG IDs was uploaded to the Pathway Analysis tool of MetaboAnalyst and a list of significant pathways was retrieved using the following parameters: Rattus norvegicus pathway library, hypergeometric test and relative-betweenness centrality.

### Bioinformatics and statistics

The normalized data matrix with ion counts of annotated metabolites was used for subsequent analysis in R. For the calculation of principal components analysis (PCA), the dudi.pca function from the CRNA package ade4 was used. A colored correlation matrix and hierarchical clustering heatmap were used to visualize the maximal metabolic alterations between each group. For the correlation matrix, the Pearson correlation coefficients were calculated for median values of metabolite abundance using R version 3.3.2., while for hierarchical clustering fold change (FC) based on metabolite abundance values were used. In the hierarchical clustering heatmap, positive values indicate the level a specific metabolite is increased, while negative values indicate the amount a specific metabolite is decreased. The LC-MS abundance data set was imported into R software for heatmap generation. Pathway activity heatmaps were generated for metabolites with intensity fold change over NX + Glc were greater than 1.3 (upregulated pathways) or lower than −1.3 (downregulated pathways). The respective metabolites were selected for pathway enrichment analysis (MetaboAnalyst, www.metaboanalyst.ca) to calculate -Log10(P) for each covered metabolic pathway. Venn diagrams were then generated for these pathway specific lists using InteractiVenn^30^. All results are expressed as mean ± SD. Statistical significance was either assessed using unpaired student’s t-test or one-way ANOVA for comparison within one group and two-way ANOVA for comparison between different groups using GraphPad Prism 7 software (La Jolla, CA). Bonferroni’s post-hoc test was used for all analyses. A p-value below 0.05 was considered significant.

## Results

### Metabolome compositions differ strongly for both cell type and insult severity

**Figure 1:**
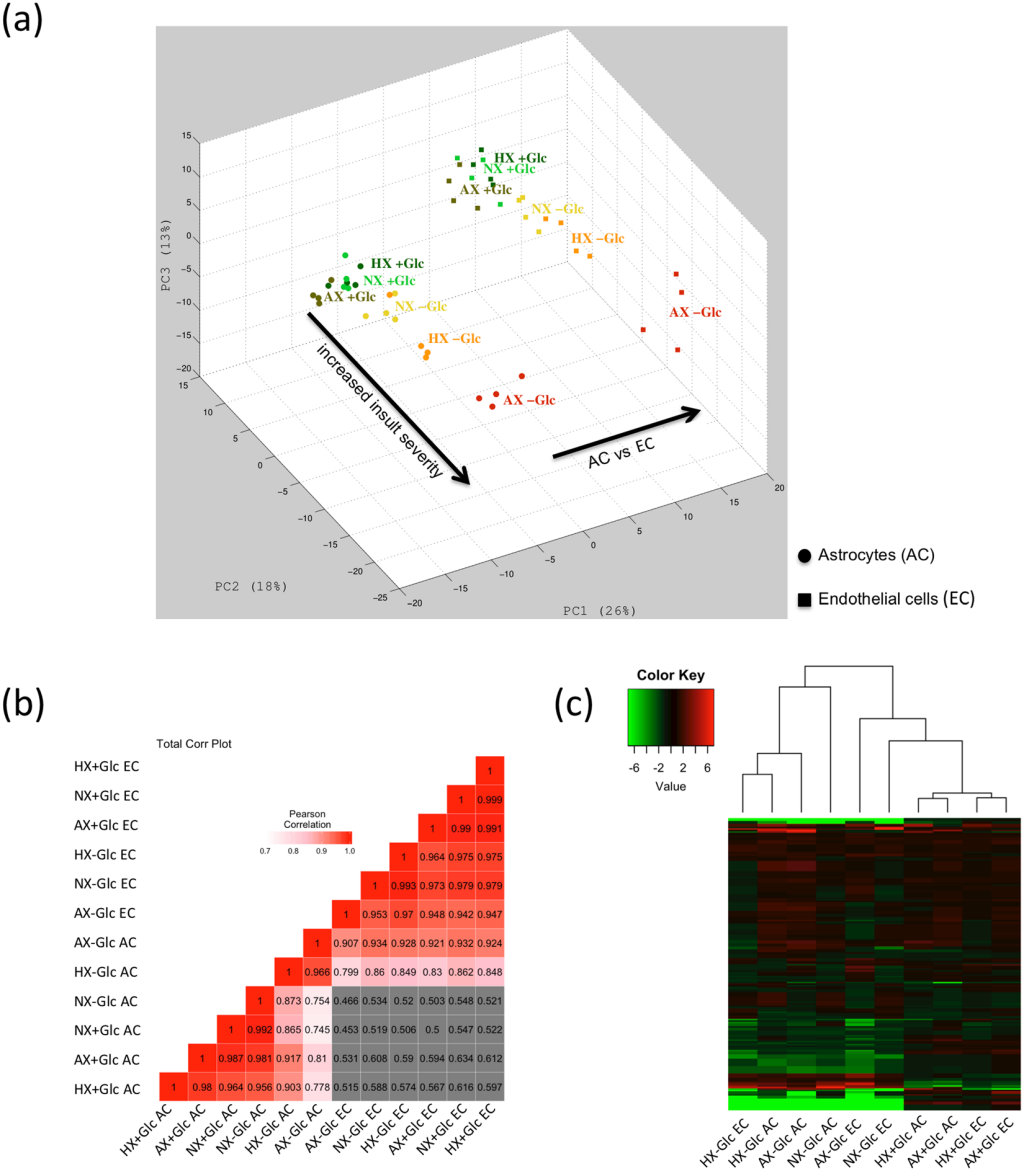
Metabolome compositions differ strongly for both cell type and insult severity. **(a)** Principal component analysis of the metabolomes of AC (circles) and EC (squares) exposed to normoxia, hypoxia and near anoxia in presence and absence of glucose. Axes represent the first three principal components (PC) of the dataset and their contribution to overall metabolome variance in percent (%). (**b)** Correlation matrix plot of all AC and EC conditions. The Pearson correlation coefficients were calculated by log2 transformed ratios of the median values of fold changes and are represented by gradient colors as indicated in the color key. Correlations less than 0.6 are shown in gray. **(c)** Hierarchical clustering heatmap of different injury conditions in AC and EC samples. Metabolites significantly decreased are displayed in green and those significantly increased displayed in red. The brightness of each color corresponds to the magnitude of the difference when compared with average values. Normoxia (NX), hypoxia (HX) and near anoxia (AX) in presence and absence of glucose (±Glc). To enable comparison of data between the cell types, the metabolite intensities were normalized to total ion counts. n = 4.

Using an untargeted LC-MS based approach we compared the metabolic profiles of primary rat brain microvascular endothelial cells (EC) and astrocytes (AC) after exposure to normoxia, hypoxia or near anoxia for 24 h in glucose containing media or glucose-free media (to simulate ischemia *in vitro*). With a combination of known standard retention times and accurate mass matching to the KEGG library 372 metabolites were annotated, allowing a global overview of cellular metabolism. Principal component analysis (PCA) was first performed to assess overall metabolome differences between the groups. Figure 1a shows graphical representation of PCA scores for all treatments of both AC (circles) and EC (squares). The plot shows the four biological replicates clustering closely together implying good reproducibility of the observed changes (Fig. 1a). The axes represent the major principal components (PC) of the dataset defining the directions of the highest variance/difference i.e. where the metabolome is most different. Distinct separation of AC versus EC data points on the PC1 axis shows the cell type makes the largest contribution to metabolome composition. Insult severity (PC2 axis) is the second most important parameter affecting metabolome composition. Close clustering of the O_2_ deprivation groups for both cells on PC2 indicates that only small changes in the metabolome occur during insult. In contrast oxygen-glucose deprivation (OGD) induces a spread of the different exposures along the second axis demonstrating more profound changes (Fig. 1a). Pearson correlation matrix confirmed the similarities between AC and EC under different injury conditions and shows the different cell types have different metabolic constitutions (Fig. 1b). To further assess these changes, a hierarchical clustering heatmap was constructed comparing the different AC and EC metabolic compositions to their own baseline conditions (NX + Glc). Clustering of conditions within the hierarchical tree shows similar dynamic behaviour and emphasizes that glucose deprivation dramatically alters metabolite abundance (Fig. 1c). Thus AC and EC metabolomes are quite different under all conditions and, surprisingly, sole oxygen deprivation is not a strong modulator of metabolome composition.

### Baseline metabolomic profiles: AC have prominent sugar metabolism whereas EC exhibit strong purine and amino acid metabolism

**Figure 2:**
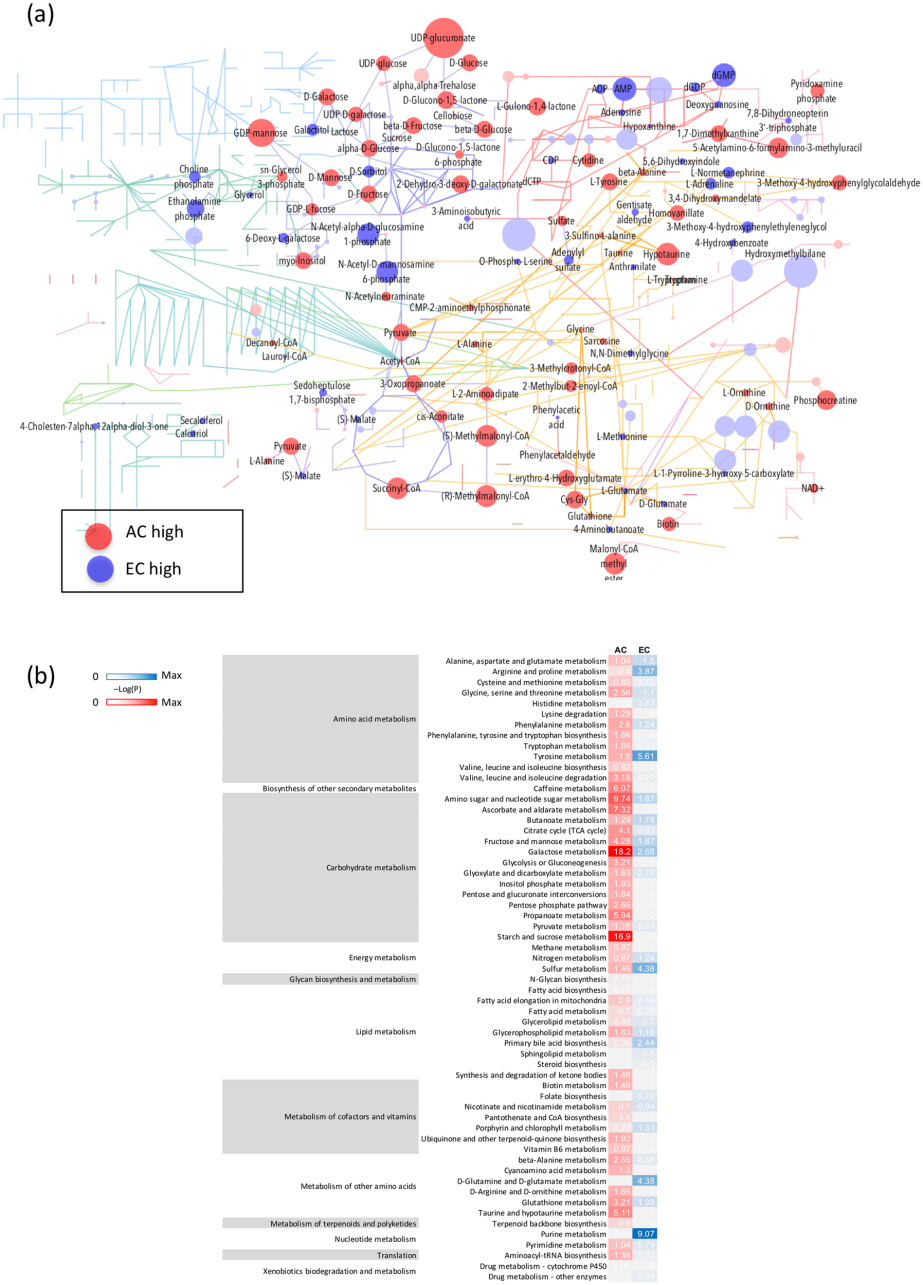
Baseline metabolomic profiles: AC have prominent sugar metabolism whereas EC exhibit strong purine and amino acid metabolism. The relationship of metabolite abundance between AC and EC during baseline conditions (NX + Glc) using two different pathway analyses. (**a)** Overview of metabolites highly abundant in AC (red) and EC (blue) embedded in their metabolic pathways using MetaboAnalyst (v.4.0) and KEGG metabolic pathways tool. The size of the circles represents the fold change with dark and light colors depicting significant and non-significant differences respectively. Purple lines represent carbohydrate metabolism, red lines nucleotide metabolism, dark green lines lipid metabolism, orange: amino acid metabolism. n = 4. (**b)** Heatmap shows the major metabolic pathway activity in different cell types, AC (red) and EC (blue), relative to each other. The -Log10(P) value for each pathway was calculated using the MetaboAnalyst v.4.0 enrichment analysis tool. n = 4.

To identify differentially activated pathways in EC and AC, we determined the main metabolites accounting for cell type specific differences under normoxic conditions. From a comprehensive list of all detected metabolites, the ratio of metabolite abundance of AC to EC was calculated for baseline conditions (NX + Glc). Fold changes and p-values were mapped on the global metabolism KEGG pathway map (Fig. 2a). Metabolites increased in AC compared to EC are shown in red circles whereas metabolites higher in EC are blue. The circle size reflects the fold change with dark colors indicating significant alterations and light colors insignificant ones. The map shows that many AC metabolites are expressed at significantly higher levels than EC, particularly in pathways that account for carbohydrate metabolism and TCA cycle (Fig. 2a). In contrast, EC accumulate metabolites involved in nucleotide and lipid metabolism. To further characterize the differentially active pathways in the two cell types enrichment analysis was performed using the MetaboAnalyst tool and -Log10(P) values were calculated. The data is presented in a heatmap that depicts the major active pathways in AC and EC in relation to each other (Fig. 2b). Correlating well with the KEGG analysis results we again identified pathways linked to carbohydrate metabolism from top hits for AC including galactose, starch and sucrose, amino sugar and nucleotide sugar and fructose metabolism, as well as glycolysis and citrate cycle. In contrast, EC metabolites of the purine metabolism pathway as well as different amino acid pathways such as tyrosine, D-glutamine/D-glutamate and arginine/proline metabolism were more overrepresented compared to AC. In summary AC had high levels of metabolites related to sugars and activated sugars compared to EC, whereas EC nucleotides and select amino acid metabolites were more abundant in relation to AC.

### Energy capacity during normoxic conditions in AC and EC

**Figure 3:**
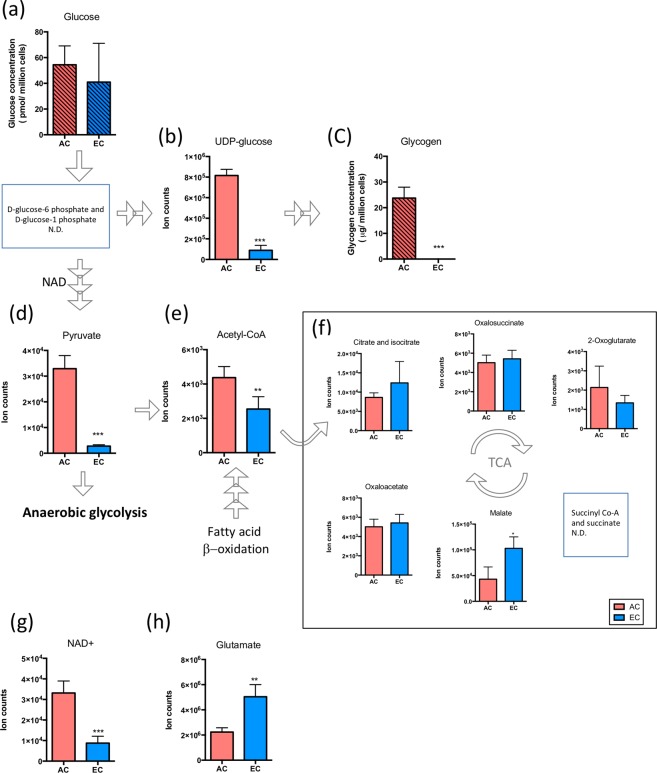
Energy capacity during normoxic conditions in AC and EC. Levels of metabolites that participate in glycogen synthesis; glucose (**a**), UDP-glucose (**b**) and glycogen (**c**). Histograms with hatched pattern show cellular glucose and glycogen levels detected by specific and independent quantitative biochemical analysis. The levels of pyruvate (**d**) and abundance of metabolites in TCA cycle (**e, f**). Levels of NAD + a co-factor of glycolysis (**g**) and glutamate (**h**), another carbon source of the TCA cycle. EC compared to AC under baseline conditions (NX + Glc). n = 4. *P < 0.05, **P < 0.01, ***P < 0.001; unpaired student’s t-test. N.D. not determined. Mean ± SD. n = 4.

To better understand the individual cellular capacity for energy generation we compared the levels of different metabolites involved in glucose metabolism. Since the LC-MS could not distinguish molecules with the same molecular formula such as hexoses (fructose, galactose and glucose), we used independent assays to accurately measure glucose and glycogen levels. Although similar amounts of glucose were detected in AC and EC (Fig. 3a), AC maintained large glycogen stores whereas EC clearly did not (Fig. 3c). In agreement higher levels of UDP-glucose, the major glycogen building block, were observed in AC (Fig. 3b). Although D-glucose-6 phosphate and D-glucose-1 phosphate were not detected specifically, these results suggest a relatively high rate of AC glycogen biosynthesis. Moreover pyruvate and acetyl-CoA levels were elevated in AC (Fig. 3d,e) and the glycolysis and TCA cycle co-factor NAD^+^ was highly abundant in AC (Fig. 3g). Intriguingly, most detected TCA related metabolites showed similar levels in both cell types - an exception being significantly higher levels of malate (Fig. 3f) and glutamate (Fig. 3h), other TCA cycle carbon sources, in EC. This suggests that under baseline conditions TCA cycle activity is similar in both cells whereas glycogenesis and glycolysis is more predominant in AC. Additional flux analysis will provide more detailed insight into cell-specific central carbon metabolism activity.

### Injury modulates the AC and EC metabolome differently

**Figure 4:**
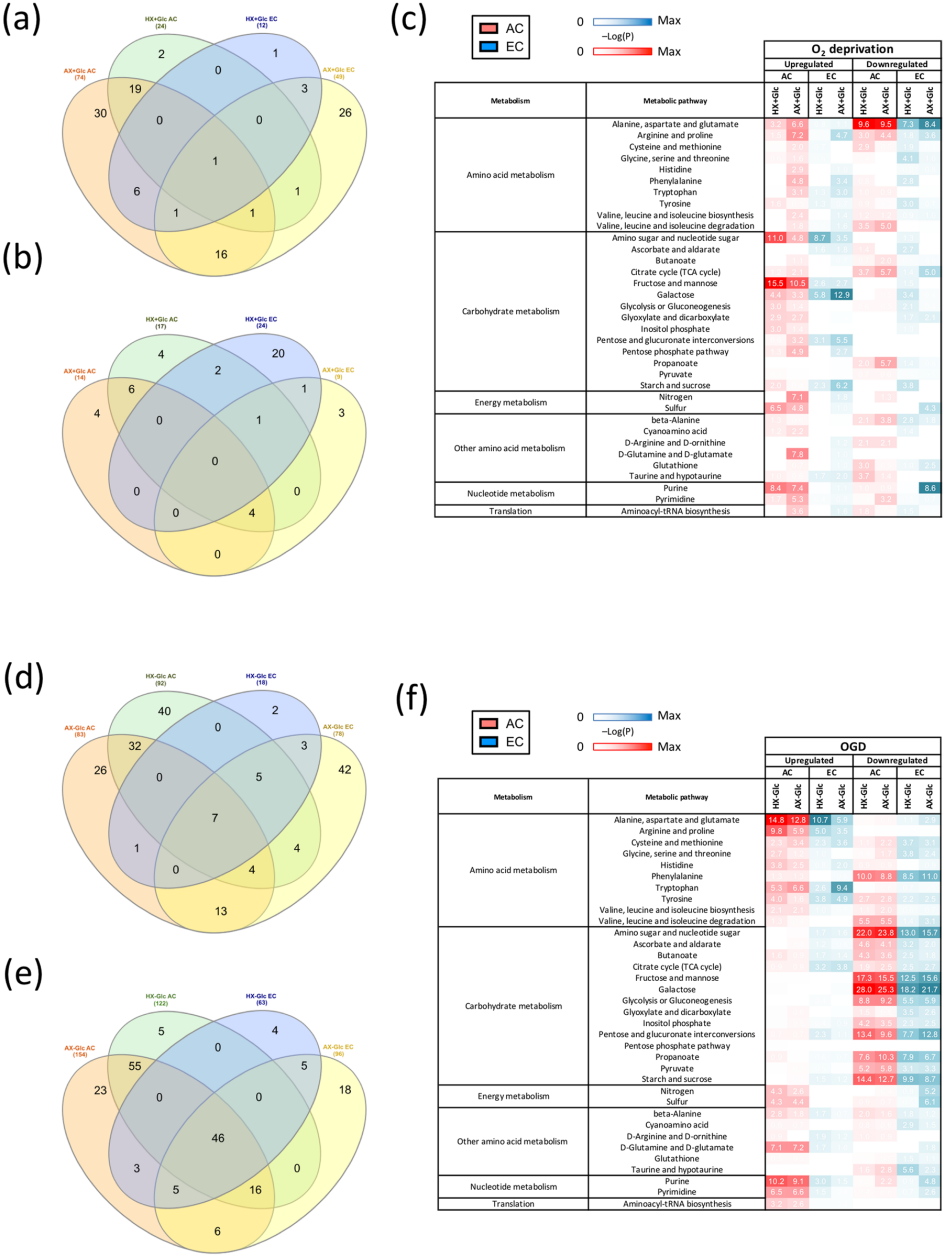
Injury modulates the AC and EC metabolome differently. Venn diagrams show correlations of increased (**a,d**) and decreased (**b,e**) metabolites between AC and EC after 24 h hypoxic (HX + Glc) and anoxic (AX + Glc) stress conditions and during 24 h oxygen-glucose deprivation (HX-Glc) and (AX-Glc). (**c,f**) Heatmaps show comparison of differentially activated metabolic pathways (up and down regulated) after oxygen deprivation (**c**) and OGD (**f**) in AC (red) and EC (blue). Metabolites were considered increased or decreased based on the intensity fold change (upregulated pathways greater than 1.3 or downregulated pathways lower than −1.3) compared to NX + Glc. The –Log10(P) was calculated using the MetaboAnalyst v.4.0 enrichment analysis tool. n = 4.

Enrichment analysis was next used to obtain a global overview of metabolic changes during O_2_ deprivation. Venn diagrams show the correlations of altered metabolites between different degrees of O_2_ deprivation in the two cell types (Fig. 4a,b). Overall, during O_2_ deprivation 107 metabolites were increased in AC and EC, whereas 45 compounds were decreased. In the increased part, AX + Glc caused a stronger induction compared to HX + Glc (Fig. 4a). Furthermore, in AC relatively more metabolites were elevated during O_2_ deprivation, namely 77 metabolites in AC versus 56 in EC (Fig. 4a). In contrast, EC had more decreased metabolites compared to AC (31 versus 21 metabolites respectively). Interestingly overall more compounds were decreased during HX + Glc than AX + Glc in EC (Fig. 4b), whereas there was more overlap of altered metabolites in AC (Fig. 4a,b). An overview of the pathways enriched in EC and AC under AX + Glc generated using KEGG analysis confirmed many more metabolites were altered in AC (Fig. S2). The heatmap presents the most enriched pathways in the two cell types under O_2_ deprivation (Fig. 4c). Particularly, carbohydrate metabolism enrichment was relatively higher in AC under mild oxygen deprivation (HX + Glc) compared to EC. Furthermore, severe oxygen deprivation (AX + Glc) correlates with enriched amino acid, energy and nucleotide metabolism in AC. Pathway enrichment changes during O_2_ deprivation were restricted to only a few different pathways in EC, pointing to a more rigid and less versatile metabolic profile compared to AC.

More severe injury conditions were mimicked by combined oxygen and glucose deprivation (OGD). In AC 132 metabolites were increased compared to only 81 in EC (Fig. 4d). More metabolites were decreased during OGD compared to sole O_2_ deprivation, 157 compounds in AC versus 103 compounds in EC (Fig. 4e). The KEGG analyses maps presented in Fig. S3 provide a visual overview of the metabolites most affected by OGD in both cells. Together with the heat map in Fig. 4f a dramatic reduction of carbohydrate-related pathways is evident in OGD as expected (Figs. 4f and S3). Only amino acid metabolism showed an accumulation of metabolites in both cell types. Interestingly, energy and nucleotide metabolism pathways as well as the glutamine/glutamate pathway were significantly increased under OGD in AC compared to EC (Fig. 4f). Translation metabolism was also elevated in AC albeit at comparatively lower levels (Fig. 4f). In accordance with O_2_ deprivation data, we again noted that increasing insult severity results in more profound metabolic changes. Taken together, during stress conditions differential and distinct metabolic changes occur that probably directly impact adaptation, cell behaviour and survival.

### Glycogenolysis and glycolysis support AC and EC energy consumption

**Figure 5:**
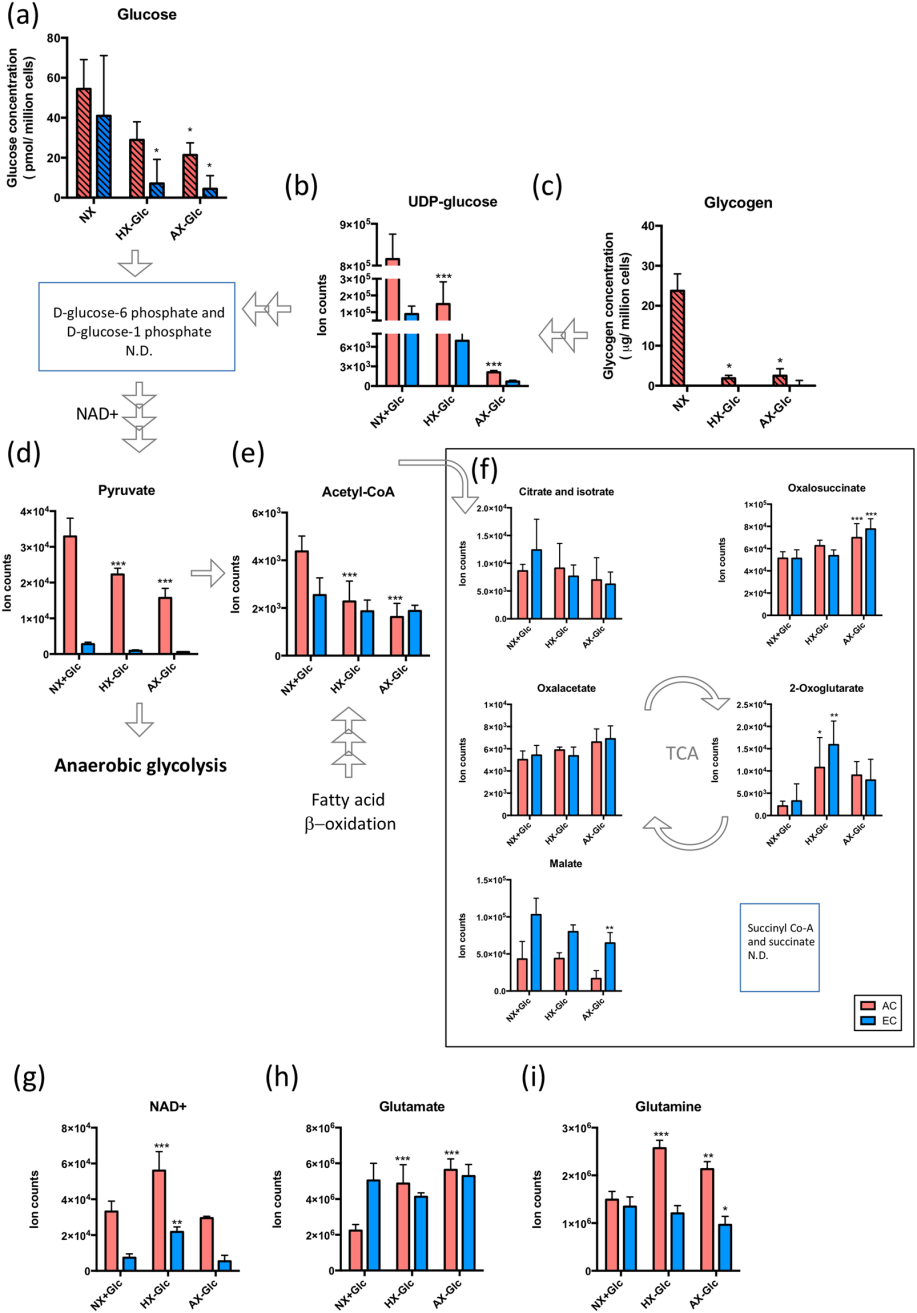
Glycogenolysis and glycolysis support AC and EC energy generation differently. AC and EC levels of metabolites that participate in glycogen synthesis; glucose (**a**), UDP-glucose (**b**) and glycogen (**c**). Hatched histograms highlight metabolites detected by independent and specific quantitative biochemical analysis. The levels of pyruvate (**d**) and abundance of metabolites in TCA cycle (**e,f**). Levels of NAD + a co-factor of glycolysis (**g**), glutamate (**h**) and glutamine (**i**), other carbon sources of the TCA cycle. AC and EC were exposed to normoxia (NX), hypoxia (HX) and near anoxia (AX) without glucose (-Glc) for 24 h. All conditions compared to their own control conditions (NX + Glc). N = 4. *P < 0.05, **P < 0.01, ***P < 0.001; 1-way ANOVA. N.D. not determined. Data presented as mean ± SD of n = 4.

Since strong changes in glycogen/glycolysis metabolism were observed, we further scrutinized the cell-specific mechanisms of cellular energy generation during OGD. Accurate measurement of glucose and glycogen by specific kits were used. Unsurprisingly, OGD exhausted cellular glucose levels in both cells although residual levels (20–30% of baseline) were always maintained in AC (Figs. 5a and S4a). Whereas AC glycogen levels increased during O_2_ deprivation, OGD completely depleted all glycogen stores (Figs. 5c and S4b). Glycogen was not detectable under any condition in EC (Figs. 5c and S4b). UDP-glucose levels decreased in a severity-dependent manner for both cell types (Fig. 5b). Downstream of glucose metabolism the considerably low normoxic EC pyruvate levels were also rapidly depleted but AC pyruvate concentrations, although decreased by OGD, remained at 50–60% (Fig. 5d). In contrast acetyl-CoA levels were similar although surprisingly OGD significantly decreased AC stores whereas EC levels remained constant (Fig. 5e). Despite these observations the data showed TCA cycle intermediates during OGD were comparable in both cells. Levels of oxalosuccinate and 2-oxoglutarate similarly increased in both cell types during OGD correlating with the induction of NAD + (Fig. 5f,g). Interestingly, malate levels were decreased in EC during severe OGD without a change in oxalacetate levels, indicating that malate levels may contribute to some other pathway (Fig. 5f). Despite reduced Acetyl-CoA levels a sharp increase in glutamate and glutamine observed solely in AC (Fig. 5h,i) probably contributing to TCA cycling.

### AC have a greater adaptive capacity to face environmental stress

**Figure 6:**
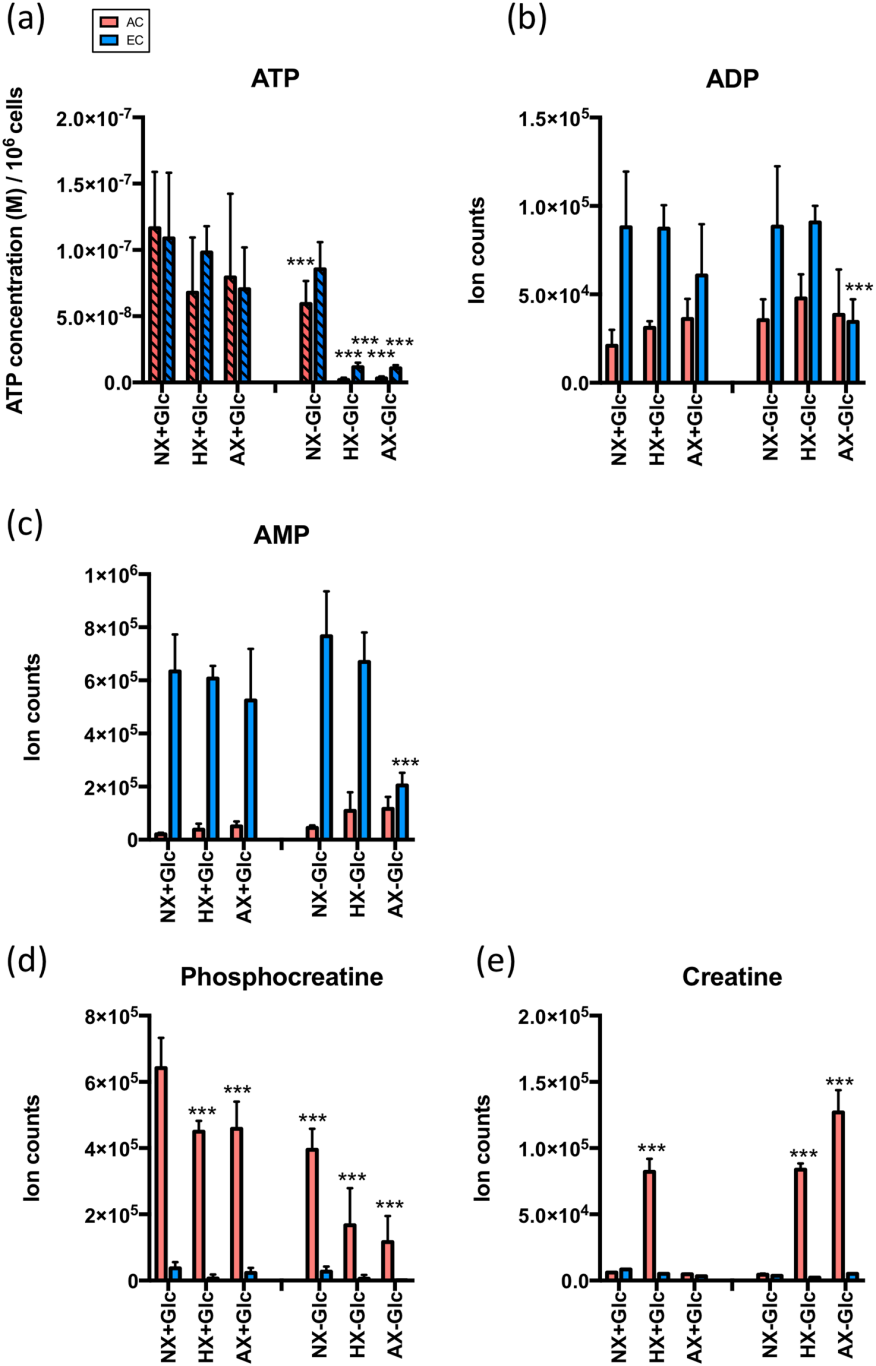
AC have a greater adaptive capacity to face environmental stress. Modulation of cellular ATP concentrations under different conditions. Abundance of ATP cycle-related metabolites, ADP (**b**) and AMP (**c**) under different conditions. d,e Amount of creatine (**d**) and phosphocreatine (**e**) under different conditions. AC and EC were exposed to normoxia (NX), hypoxia (HX) and near anoxia (AX) with or without glucose (±Glc) for 24 h. Data compared to baseline conditions (NX + Glc) in each cell type. The ATP data presented as mean ± SD of n = 3 (in EC) and n = 7 (in AC) independent experiments, and the LC-MS data showed as mean ± SD of n = 4 biological replicates. *P < 0.05, **P < 0.01, ***P < 0.001; 1-way ANOVA.

Finally we investigated if ATP associated metabolites could account for the differential sensitivity of AC and EC to O_2_ deprivation and OGD as we and others previously observed^31–33^. Accurate measurement of ATP levels was performed with an independent assay. Compared to normoxia ATP levels were largely maintained during oxygen deprivation in both cell types (Fig. 6a). Increasing ischemia however strongly depleted ATP levels illustrating a reduced capacity to replenish stores from alternative energy sources. Notably, EC levels of ADP and particularly AMP were constantly much higher than AC in all conditions (Fig. 6b,c). We further compared the abundance of metabolites involved in creatine biosynthesis and creatine (CR) /phosphoCR (pCR) cycle since pCR generation can also stabilize cytosolic ATP^34^. In general levels of glycine, ornithine, and S-adenosyl-L-methionine were up to 2 times higher in AC than EC, suggesting a consistently stronger rate of CR biosynthesis (Fig. S5). Supporting this assumption pCR levels were also consistently and significantly elevated in AC compared to EC, and remained impressively high even after OGD (Fig. 6d). Intriguingly, similarly low CR levels were apparent in both cells during normoxia but most injury conditions strongly induced CR in AC, again suggesting its use in replenishing ATP pools. In contrast pCR levels were almost undetectable in EC (Fig. 6e) indicating they don’t use pCR to support their energy requirements.

## Discussion

**Figure 7:**
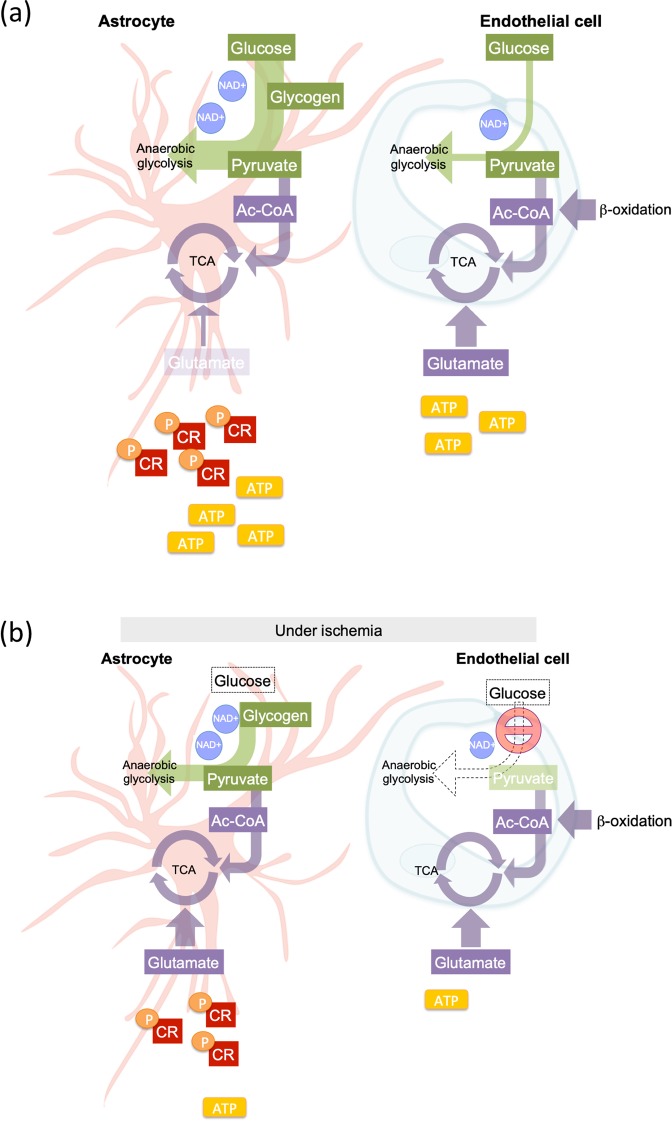
Schematic overview of energy generation by AC and EC. We propose the following cellular models of energy generation; (**a)** Under normoxic/resting conditions, AC predominantly use glucose to support glycogenesis and anaerobic glycolysis (green) as well as the TCA cycle (purple) via pyruvate synthesis. Brain EC sparingly utilize glucose directly to support glycolysis, but glutamate and/or β-oxidation likely contribute to EC TCA cycle to maintain the central carbohydrate metabolism. (**b)** During injury conditions, elevated glycogen and pyruvate reserves, pCR as well as excellent antioxidant capacity in AC demonstrate a high metabolic flexibility. Comparatively EC are more metabolically rigid, attempting to sustain their resting/baseline profile at almost all cost. Arrow thickness represents the degree of pathway activity. The number of repeated circles (NAD + ) and rectangles (ATP and CR) indicate the metabolite level. Metabolite abundance is indicated by rectangle transparency with dark and light depicting high and low levels respectively under different conditions. The dotted arrow indicates inactivated pathways.

As metabolic dysregulation plays a prominent role in many brain and vascular-related pathological processes associated with BBB disturbance, we hypothesized that differential cell-specific metabolomic adaptation majorly influences barrier functionality. This is the first study to directly profile and compare the metabolomes of brain EC and AC cells during normoxia and increasingly severe injury conditions. Despite sharing some features, EC and AC exhibited distinct normoxic metabolic signatures overall. We noted that AC rapidly respond to environmental stress whereas EC constantly attempt to maintain baseline barrier characteristics. In particular, glycolysis and TCA cycle analyses revealed distinct cell-specific metabolomic features and functions in different conditions. A schematic overview of energy generation in each cell is shown in Fig. 7.

### Differential normoxic metabolic features of AC and brain EC

Published data suggests that all non-brain EC exhibit similar metabolic profiles, being highly glycolytic and reliant on β-fatty acid oxidation to support their energy usage and balance redox homeostasis^8,9^. We observed the brain EC metabolomic composition to be quite different to non-brain EC. Under baseline normoxic conditions nucleotide, amino acid, sulfur as well as purine metabolism were highly altered in brain EC. In contrast to non-brain EC carbohydrate metabolism and β-fatty acid oxidation was not highly activated. This differential metabolism correlates with the specialized function of the brain endothelium. Since glucose is critical for brain function, we suggest that brain EC maintain low glucose consumption (glycolysis) to facilitate effective transport across the endothelium. ECs may use glutamate as an alternative fuel to sustain TCA cycling and amino acid metabolism. Indeed, brain EC are resistant to glutamate-induced cytotoxicity^35^ and a highly-activated glutamate metabolism was proposed to help survive their special tissue environment and perform their BBB function^36,37^. This agrees nicely with the fact that we detected higher amounts of glutamate in EC compared to AC. Brain EC also exhibited strong purine metabolism, presumably to support nucleotide and protein synthesis^38–40^ and perhaps maintain continuous high expression of membrane transporters and tight junction proteins. The AC metabolome was comparatively highly active during normoxic conditions with a predominance of glycolytic and central carbon metabolites as observed in previous studies^22^. Levels of pyruvate and the glycolysis co-factor NAD^+^ were higher in AC, but acetyl-CoA levels were similar to EC suggesting most of the pyruvate may contribute to anaerobic glycolysis, lactate and energy generation in line with observations by others^17^. Large amounts of glycogen, a readily mobilized form of glucose, were measured in AC as expected^17,41^, but never detected in brain EC. As the highly sensitive isotopomer analyses was needed to measure HUVEC levels^42^, it seems unlikely glycogen is used by EC.

From our data it seems plausible that metabolic interaction between AC and EC is similar to that reported for AC and neurons. Indeed high AC glycogen stores and related metabolites mean paracrine energy shuttling could significantly benefit all surrounding cells. As the major anti-oxidant producer in the brain, AC also have comparatively high basal levels of glutathione (GSH) and NAD^+^ which are essential for brain redox homeostasis^20^ and β-alanine that boosts GSH synthesis^43^. We did not utilize co-cultures in this study but our observations nevertheless suggest AC and EC likely work together in union. A division of labor with EC particularly focused on barrier formation and molecular transportation, and AC supporting nutrient/energy and redox balance would facilitate optimal functioning of both the barrier and NVU. Studies investigating such possibilities are now underway.

### During injury AC dynamically modulate their metabolism whereas brain EC are less flexible

Prolonged oxygen deprivation significantly impairs BBB stability but perivascular cells are known to have higher tolerance to hypoxia than brain EC^31–33^. Interestingly even mild oxygen deprivation (HX + Glc) altered the metabolomic composition of both EC and AC, albeit differentially. Surprisingly, brain EC glycolytic pathways remained unchanged during injury conditions suggesting they preferentially spare energy consumption in contrast to non-brain EC that induce glycolysis to facilitate energy replenishment during proliferative processes and hypoxia-induced angiogenesis^9,12,44^ - an intriguing fundamental difference. During AX + Glc, EC purine metabolites were further decreased suggesting limited investment in DNA and protein synthesis to compensate reduced energy availability. In comparison, AC exhibited a much more dynamic response. Oxygen deprivation decreased amino acid and TCA related metabolites in correlation with increased glycolytic metabolism as previously shown^45,46^. Highly elevated nitrogen, sulfur and nucleotide metabolism was also observed. Thus despite becoming more glycolytic AC retain the capacity to mobilize energy consuming pathways.

Unsurprisingly, levels of carbohydrate-related metabolites declined in both cells during OGD. However the particularly striking decrease across the EC metabolome in correlation with increasing injury severity suggest a progressive functional decline. Indeed significantly reduced nitrogen, sulfur and nucleotide metabolism was suggested to preempt the onset of cell death^47,48^. In contrast, OGD dramatically elevated select AC amino acid, sulfur, nitrogen and nucleotide metabolites in line with induction of non-carbohydrate pathways to support energy generation during severe stress^49^. AC also boosted glycolysis pathways while EC preferentially maintained TCA cycling. As glycogen is rapidly consumed during glucose deprivation^50^ pyruvate is likely a major contributor to AC anaerobic glycolysis under ischemic conditions^18,51^. Notably, AC pyruvate levels always remained dramatically higher than those of EC, an observation in line with our “glucose-sparing” hypothesis for the brain endothelium. Glucose withdrawal might also push AC to utilize more glutamate (increase its turnover) as an alternative carbon source as observed in glioma cells^52^. Increased glutamate and glutamine-related metabolites, and maintenance of both carbohydrate (glycolysis and TCA cycle) and amino acid levels, agrees with such responses supporting ischemic AC metabolic homeostasis^53^. Intriguingly, and perhaps counter-intuitively, levels of TCA cycle metabolites were similar in both cells. Indeed despite very low pyruvate levels and ongoing stress, brain EC acetyl-CoA and TCA related metabolites were constantly maintained at baseline normoxic levels. It is very tempting to speculate that β-fatty acid oxidation and amino acid metabolism contribute to EC acetyl-CoA synthesis during ischemia. Interestingly, driving the TCA cycle in such a way would bring the brain EC metabolic profile closer to that of resting/quiescent peripheral EC. Strikingly, a very recent study showed that diseases associated with BBB disruption exhibit a core transcriptomic dysfunction module that shifts brain ECs into a peripheral EC-like state^54^. Integrating such transcriptomic and metabolomic data will provide unprecedented insight into potential ways to modulate BBB. Taken together it seems BBB EC are metabolically distinct from non-brain EC by constantly resisting environmental stress to sustain their “physiological” characteristics - a feature to preserve barrier homeostasis as long as possible. This largely inflexible and stoic profile completely contrasts with the intrinsic dynamic diversity of the AC metabolome.

### Creatine/phosphocreatine cycling correlates with AC ability to tolerate severe stress conditions

Creatine (CR) is essential to rapidly replenish ATP in high energy-expending organs such as brain. Phosphocreatine (pCR) depletion and induction of its associated pathway correlated with injury severity highlighting that compensatory shifts in this phospho-transfer network likely safeguard AC energetic homeostasis and is an important resource^55,56^. As CR and pCR levels were almost undetectable in EC it was unexpected that cytosolic ATP levels were always comparable in both cells. A CR kinase knockout mouse also maintained normal ATP levels^57^ showing CR/pCR may not always contribute to intracellular ATP pools *per se*. Presumably high levels of AMP, acetyl-CoA and TCA cycle metabolites in brain EC supports energy generation similar to non-brain EC mechanisms^58^. Consistently elevated ADP and AMP levels and activation of high-energy requiring pathways, even in baseline conditions, confirmed brain EC are high-energy consumers compared to AC. As maintaining the large number of EC mitochondria is surely a major energy drain, the dramatic decrease in AMP and ADP levels during severe OGD is probably partially provoked by cell stress from constant mitochondrial activity^59^. Thus despite their ATP turnover likely exceeding that of AC the CR/pCR system is not an available EC resource. Conversely, stressed AC gradually increase AMP and ADP levels, nicely correlating with better energy reserves and flexibility. It is highly feasible that AC-driven CR secretion benefits brain vascular function as observed between AC and neurons^34^.

This study provides the first detailed comparison of AC and EC metabolomic profiles under different injury conditions. Overall, the distinct cell-specific portraits reflect their important roles within the NVU during brain vascular (BBB) homeostasis and disturbance. A schematic overview is provided in Fig. 7a,b. It is undeniable that more detailed insight of AC and EC processes, demands and adaptability/flexibility could provide innovative ways to modulate barrier permeability. In this regard use of increasingly severe injury conditions in this study was strategic as the degree of hypoxic and oxidative stress likely varies considerably between brain regions during disease - and thereby presumably impacts barrier cells in those regions differently. Thus while it is unclear how our injury simulations correspond to environmental conditions during disease processes *per se*, comparing different severities unveils modulations that may occur during diverse scenarios^60^. We remain mindful that cellular organization and interactions that occur *in vivo*, extracellular matrix composition as well as blood flow, are absent in this simplified model. As such it is possible the metabolomic responses observed herein will be modified *in vivo* – but which ones and the extent of modification requires further testing. Indeed whether any of these cell-specific alterations definitively contribute to changes in BBB function and EC dysfunction during disease processes remains to be tested. It will also be intriguing to explore if metabolite supplementation could have therapeutic potential in counteracting progression of such diseases. Overall, it seems poignantly clear that the extensive reserve capacity and flexible disposition of AC implies metabolic crosstalk with stressed EC is very likely. As such combining isotopic or other tracers to specifically track metabolite shuttling will be essential to fully understand how metabolomic change, and exchange, impact BBB homeostasis. However, if frequent shuttling occurs such alterations will be difficult to track *in vitro* and be even more challenging to observe *in vivo*. Prior insight of potentially relevant metabolites as presented herein will undoubtedly be valuable.

In conclusion, this comprehensive dataset is a rich resource that presents novel insight into cell-specific metabolic changes and pathways that might impact vascular stability during diverse injury scenarios.

## Supplementary information

Supplementary Figures.

## Data Availability

The datasets generated during and/or analyzed during the current study are available from the corresponding author on request.

## References

[1] Engelhardt S, Patkar S, Ogunshola OO (2014). Cell-specific blood-brain barrier regulation in health and disease: A focus on hypoxia. Br. J. Pharmacol..

[2] Delaney, C. & Campbell, M. The blood brain barrier: Insights from development and ageing. *Tissue Barriers* **5**, (2017). 10.1080/21688370.2017.1373897 PMC5788423 28956691

[3] Abbott NJ, Patabendige AAK, Dolman DEM, Yusof SR, Begley DJ (2010). Structure and function of the blood–brain barrier. Neurobiol. Dis..

[4] Zhao Y (2014). Metabolomic Heterogeneity of Pulmonary Arterial Hypertension. PLoS One.

[5] Mayr M, Madhu B, Xu Q (2007). Proteomics and Metabolomics Combined in Cardiovascular Research. Trends Cardiovasc. Med..

[6] Park KS, Xu CL, Cui X, Tsang SH (2018). Reprogramming the metabolome rescues retinal degeneration. Cell. Mol. Life Sci..

[7] Vandekeere S (2015). Role of Endothelial Cell Metabolism in Vessel Sprouting. Cell Metab..

[8] Kalucka, J., Bierhansl, L., Vasconcelos, N., Li, X. & Fendt, S.-M. Quiescent Endothelial Cells Upregulate Fatty Acid β-Oxidation for Vasculoprotection via Redox Homeostasis. *Cell Metab*. **28**, (2018). 10.1016/j.cmet.2018.07.016 30146488

[9] De Bock K (2013). Role of PFKFB3-driven glycolysis in vessel sprouting. Cell.

[10] Kim B, Li J, Jang C, Arany Z (2017). Glutamine fuels proliferation but not migration of endothelial cells. EMBO J..

[11] Kim JW, Tchernyshyov I, Semenza GL, Dang CV (2006). HIF-1-mediated expression of pyruvate dehydrogenase kinase: A metabolic switch required for cellular adaptation to hypoxia. Cell Metab..

[12] Haseloff RF (2006). Differential protein expression in brain capillary endothelial cells induced by hypoxia and posthypoxic reoxygenation. Proteomics.

[13] Li, L. *et al*. An angiogenic role for the α5β1 integrin in promoting endothelial cell proliferation during cerebral hypoxia. *Exp. Neurol*. 10.1016/j.expneurol.2012.06.005 (2012). 10.1016/j.expneurol.2012.06.005 PMC3748139 22721769

[14] Hellsten, J. *et al*. Electroconvulsive seizures induce angiogenesis in adult rat hippocampus. *Biol. Psychiatry*10.1016/j.biopsych.2005.05.023 (2005). 10.1016/j.biopsych.2005.05.023 16043138

[15] Ahmad AA, Taboada CB, Gassmann M, Ogunshola OO (2011). Astrocytes and Pericytes Differentially Modulate Blood—Brain Barrier Characteristics during Development and Hypoxic Insult. J. Cereb. Blood Flow Metab..

[16] Abbott NJ (2002). Astrocyte-endothelial interactions and blood-brain barrier permeability. J. Anat..

[17] Suzuki A (2011). Astrocyte-Neuron Lactate Transport Is Required for Long-Term Memory Formation. Cell.

[18] Swanson RA, Choi DW (1993). Glial glycogen stores affect neuronal survival during glucose deprivation *in vitro*. J. Cereb. Blood Flow Metab..

[19] Dienel GA, Cruz NF (2015). Contributions of glycogen to astrocytic energetics during brain activation. Metab. Brain Dis..

[20] Dringen R, Pfeiffer B, Hamprecht B (1999). Synthesis of the Antioxidant Glutathione in Neurons: Supply by Astrocytes of CysGly as Precursor for Neuronal Glutathione. J. Neurosci..

[21] Wilson JX (1997). Antioxidant defense of the brain: a role for astrocytes. Can. J. Physiol. Pharmacol..

[22] Hertz L, Peng L, Dienel GA (2007). Energy metabolism in astrocytes: High rate of oxidative metabolism and spatiotemporal dependence on glycolysis/glycogenolysis. J. Cereb. Blood Flow Metab..

[23] Chow J (2001). Astrocyte-derived VEGF mediates survival and tube stabilization of hypoxic brain microvascular endothelial cells *in vitro*. Dev. Brain Res..

[24] Coisne C (2005). Mouse syngenic *in vitro* blood-brain barrier model: A new tool to examine inflammatory events in cerebral endothelium. Lab. Investig..

[25] Fischer, D., Panse, C. & Laczko, E. *cosmiq-COmbining Single Masses Into Quantities*. (2018) doi:R package version 1.16.0, http://www.bioconductor.org/packages/devel/bioc/html/cosmiq.html.

[26] Ogata H (1999). KEGG: Kyoto encyclopedia of genes and genomes. Nucleic Acids Res..

[27] Katajamaa M, Orešič M (2007). Data processing for mass spectrometry-based metabolomics. J. Chromatogr. A.

[28] Chong, J. *et al*. MetaboAnalyst 4.0: Towards more transparent and integrative metabolomics analysis. *Nucleic Acids Res*. 10.1093/nar/gky310 (2018). 10.1093/nar/gky310 PMC6030889 29762782

[29] Shannon P (2003). Cytoscape: A software Environment for integrated models of biomolecular interaction networks. Genome Res..

[30] Heberle H, Meirelles GV, da Silva FR, Telles GP, Minghim R (2015). InteractiVenn: a web-based tool for the analysis of sets through Venn diagrams. BMC Bioinformatics.

[31] Engelhardt S, Huang S-F, Patkar S, Gassmann M, Ogunshola OO (2015). Differential responses of blood-brain barrier associated cells to hypoxia and ischemia: a comparative study. Fluids Barriers CNS.

[32] Ceruti S (2011). Oxygen-glucose deprivation increases the enzymatic activity and the microvesicle-mediated release of ectonucleotidases in the cells composing the blood-brain barrier. Neurochem. Int..

[33] Redzic ZB, Rabie T, Sutherland BA, Buchan AM (2015). Differential effects of paracrine factors on the survival of cells of the neurovascular unit during oxygen glucose deprivation. Int. J. Stroke.

[34] Béard E, Braissant O (2010). Synthesis and transport of creatine in the CNS: importance for cerebral functions. J. Neurochem..

[35] Domoki F, Kis B, Gáspár T, Bari F, Busija DW (2008). Cerebromicrovascular endothelial cells are resistant to L-glutamate. Am. J. Physiol. Regul. Integr. Comp. Physiol..

[36] DeBerardinis RJ (2007). Beyond aerobic glycolysis: Transformed cells can engage in glutamine metabolism that exceeds the requirement for protein and nucleotide synthesis. Proc. Natl. Acad. Sci..

[37] Schousboe A (2014). Glutamate and ATP at the Interface of Metabolism and Signaling in the Brain. Adv Neurobiol..

[38] Löscher W, Potschka H (2005). Blood-Brain Barrier Active Efflux Transporters: ATP-Binding Cassette Gene Family. NeuroRX.

[39] Mokgokong R, Wang S, Taylor CJ, Barrand MA, Hladky SB (2014). Ion transporters in brain endothelial cells that contribute to formation of brain interstitial fluid. Pflugers Arch. Eur. J. Physiol..

[40] Maoz BM (2018). A linked organ-on-chip model of the human neurovascular unit reveals the metabolic coupling of endothelial and neuronal cells. Nat. Biotechnol..

[41] Cruz NF, Dienel GA (2002). High glycogen levels in brains of rats with minimal environmental stimuli: Implications for metabolic contributions of working astrocytes. J. Cereb. Blood Flow Metab..

[42] Vizan P (2009). Characterization of the metabolic changes underlying growth factor angiogenic activation: identification of new potential therapeutic targets. Carcinogenesis.

[43] Mori M, Gähwiler BH, Gerber U (2002). Beta-alanine and taurine as endogenous agonists at glycine receptors in rat hippocampus *in vitro*. J. Physiol..

[44] Graven KK, Troxler RF, Kornfeld H, Panchenko MV, Farber HW (1994). Regulation of endothelial cell glyceraldehyde-3-phosphate dehydrogenase expression by hypoxia. J. Biol. Chem..

[45] Marrif H, Juurlink BHJ (1999). Astrocytes respond to hypoxia by increasing glycolytic capacity. J. Neurosci. Res..

[46] Genc S, Kurnaz IA, Ozilgen M (2011). Astrocyte-neuron lactate shuttle may boost more ATP supply to the neuron under hypoxic conditions–in silico study supported by *in vitro* expression data. BMC Syst. Biol..

[47] Tretyakov AV, Farber HW (1995). Endothelial cell tolerance to hypoxia. Potential role of purine nucleotide phosphates. J. Clin. Invest..

[48] Pollak N, Dölle C, Ziegler M (2007). The power to reduce: pyridine nucleotides – small molecules with a multitude of functions. Biochem. J..

[49] Escartin C (2007). Activation of Astrocytes by CNTF Induces Metabolic Plasticity and Increases Resistance to Metabolic Insults. J. Neurosci..

[50] Sochocka E (1994). Cell death in primary cultures of mouse neurons and astrocytes during exposure to and ‘recovery’ from hypoxia, substrate deprivation and simulated ischemia. Brain Res..

[51] Brown AM (2005). Astrocyte glycogen metabolism is required for neural activity during aglycemia or intense stimulation in mouse white matter. J. Neurosci. Res..

[52] Yang C (2015). Glutamine oxidation maintains the TCA cycle and cell survival during impaired mitochondrial pyruvate transport. Mol. Cell.

[53] Ji Y-F (2013). Upregulation of glutamate transporter GLT-1 by mTOR-Akt-NF-кB cascade in astrocytic oxygen-glucose deprivation. Glia.

[54] Munji, R. N. *et al*. Profiling the mouse brain endothelial transcriptome in health and disease models reveals a core blood-brain barrier dysfunction module. *Nat. Neurosci*. 10.1038/s41593-019-0497-x (2019). 10.1038/s41593-019-0497-x PMC6858546 31611708

[55] Kuiper JWP (2009). Local ATP generation by brain-type creatine kinase (CK-B) facilitates cell motility. PLoS One.

[56] Yager JY, Kala G, Hertz L, Juurlink BHJ (1994). Correlation between content of high-energy phosphates and hypoxic-ischemic damage in immature and mature astrocytes. Dev. Brain Res..

[57] In’T Zandt HJA (2004). Cerebral creatine kinase deficiency influences metabolite levels and morphology in the mouse brain: A quantitative *in vivo* 1H and 31P magnetic resonance study. J. Neurochem..

[58] Hardie DG, Pan DA (2002). Regulation of fatty acid synthesis and oxidation by the AMP-activated protein kinase. Biochem. Soc. Trans..

[59] Zhang DX, Gutterman DD (2007). Mitochondrial reactive oxygen species-mediated signaling in endothelial cells. Am. J. Physiol. Circ. Physiol..

[60] Engelhardt, S. Facing Hypoxia and Ischemia - Cell-Specific Signaling and Metabolism at the Blood-Brain Barrier. University of Zurich. https://www.zora.uzh.ch/id/eprint/104660/ (2014).

