# Supplementary material for: Cell-specific metabolomic responses to injury: novel insights into blood-brain barrier modulation
Source: Sci Rep. 2020 May 8;10:7760. doi: 10.1038/s41598-020-64722-w (PMC7210983; doi:10.1038/s41598-020-64722-w)

Supplementary information

Cell-specific metabolomic responses to injury: novel insights into blood-brain barrier modulation

Sheng-Fu Huang[1,2,#] Sabrina Fischer[1,3,#], Alexey Koshkin[1,2], Endre Laczko[4], David Fischer[4] and Omolara O. Ogunshola[1,2,*]

[1] Institute for Veterinary Physiology, University of Zurich. Winterthurerstrasse 260, CH-8057 Zurich, Switzerland

[2] Zurich Center for Integrative Human Physiology, University of Zurich. Winterthurerstrasse 190, CH-8057 Zurich, Switzerland

[3] Institute of Zoology, University of Basel, Vesalgasse 1, CH-4051 Basel, Switzerland

[4] Functional Genomics Center Zurich, University of Zurich, Winterthurerstrasse 190, CH-8057 Zurich, Switzerland

[#]These authors contributed equally to this work

Running title: Blood-brain barrier metabolomics

* Address correspondence to;

Omolara O. Ogunshola, PhD

Institute of Veterinary Physiology,

Vetsuisse Faculty, University of Zurich,

Winterthurerstrasse 260,

CH-8057 Zurich, Switzerland

Tel: +41 44 635 8805, Fax: +41 44 635 8932,

Email: larao@access.uzh.ch

ORCID ID: 0000-0002-1197-4914

Fig. S1: Characterization of primary AC and EC cultures.

(a) Isolation of primary rat brain AC was confirmed by high expression of GFAP and absence/low expression of PECAM-1 (EC marker), NG2 and PDGFRβ (pericyte markers). (**b**) Purity and morphology of primary rat brain microvessel EC monolayers was assessed by positive immunostaining for tight junction proteins (occludin, ZO-1 and Claudin-5) and the EC marker PECAM-1 at cell-cell borders. Undetectable GFAP, NG-2 and α-smooth muscle actin immunoreactivity confirmed the absence of AC and pericyte/smooth muscle cells in primary EC cultures. In all cases contamination was less than 5%. Cell nuclei are shown in blue (DAPI).

Fig. S2: O_2 deprivation causes only minimal changes in metabolomic composition

Overview of metabolite alterations in AC (**a**) and EC (**b**) after 24h severe oxygen deprivation (AX+Glc) as analyzed by MetaboAnalyst (v.4.0) and KEGG metabolic pathways tool. Red circles depict increased metabolites, blue circles show decreased metabolites. Circle size represents the fold change with dark and light colors depicting significant and non-significant differences respectively. Purple lines: carbohydrate metabolism, red lines: nucleotide metabolism, orange lines: amino acid metabolism, dark green lines: lipid metabolism, light green lines: terpenoids and polyketides metabolism, light blue lines: glucan biosynthesis. All deprivation conditions are compared to control baselines (NX+Glc). n=4.

Fig. S3: OGD profoundly modulates the cellular metabolome.

Overview of metabolite changes in AC (**a**) and EC (**b**) after 24h severe OGD exposure (AX-Glc) using MetaboAnalyst (v.4.0) and KEGG metabolic pathways tool. Red circles depict increased metabolites, blue circles show decreased metabolites. Circle sizes represent the fold change with dark and light colors depicting significant and non-significant differences respectively. Purple lines: carbohydrate metabolism, red lines: nucleotide metabolism, orange lines: amino acid metabolism, dark green lines: lipid metabolism, light green lines: terpenoids and polyketides metabolism, light blue lines: glucan biosynthesis. All deprivation conditions are compared to control baselines (NX+Glc). n=4.

Fig. S4: Differential cellular modulation of glycogenolysis and glycolysis

The cellular glucose (**a**) and glycogen (**b**) levels in AC (red) and EC (blue) in all different conditions. AC and EC were exposed to normoxia (NX), hypoxia (HX) and near anoxia (AX) without glucose (-Glc) for

24h. All conditions are compared to their control conditions (NX+Glc). N=4. *P<0.05, **P<0.01, ***P<0.001; 1-way ANOVA. N.D. not determined. Mean ± SD. n=4.

Fig. S5: Strong CR biosynthesis by AC but not EC

(a) Schematic depicts the metabolites involved in creatine biosynthesis. Levels of glycine (**b**), ornithine (**c**) and S-adenosyl-L-methionine (**d**) in AC and EC under injury. AC and EC were exposed to normoxia (NX), hypoxia (HX) and near anoxia (AX) with or without glucose (±Glc) for 24h. N=4. *P<0.05, **P<0.01, ***P<0.001; 1-way ANOVA compared to baseline (NX+Glc) conditions in respective cell type. Mean ± SD. n=4.

Supplemental Figs

Fig. S1. Characterization of primary AC and EC cultures

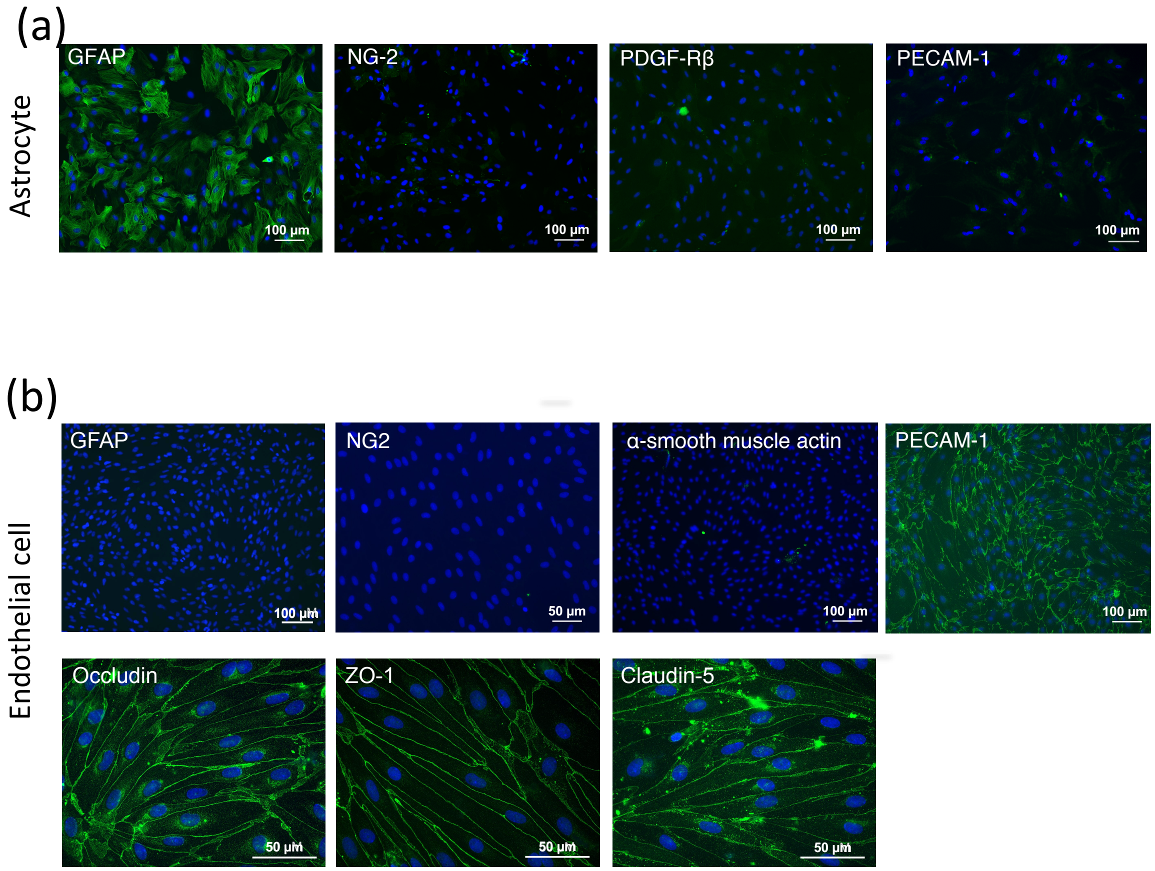

Fig. S2 O_2 deprivation causes only minimal changes in metabolomic composition

(a)

Astrocytes

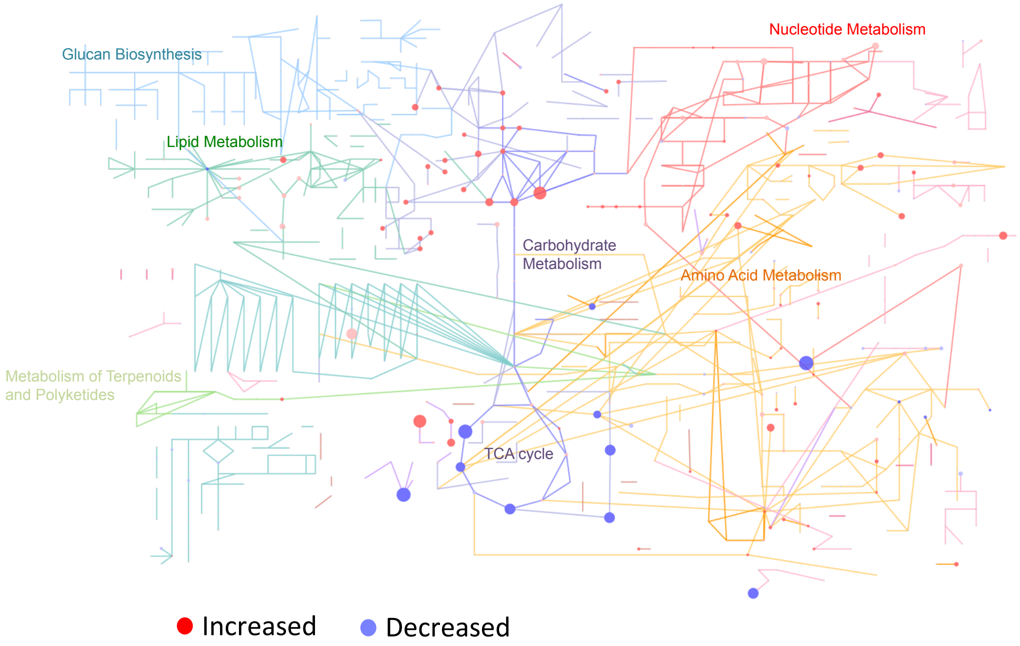

(b)

EC

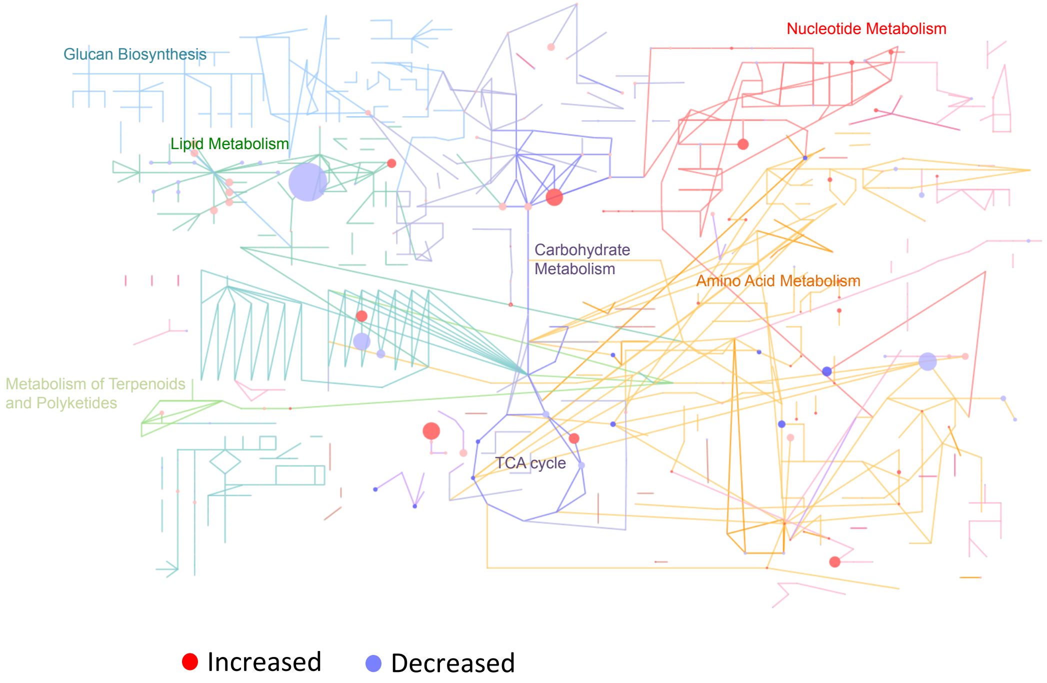

Fig. S3. OGD profoundly modulates the cellular metabolome

(a)

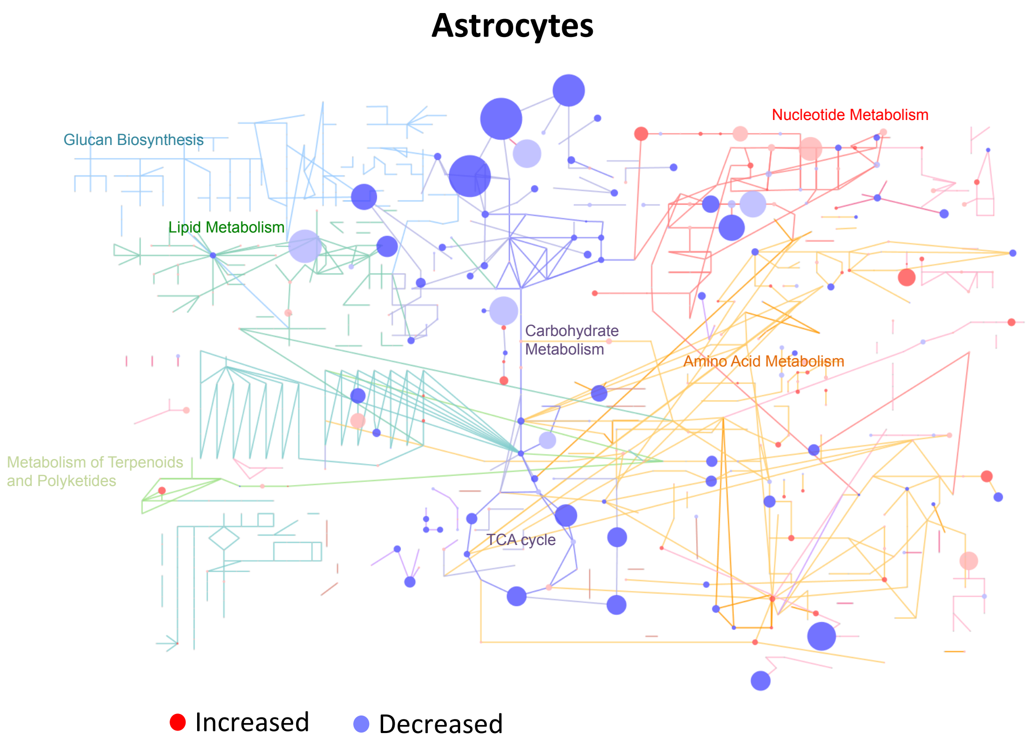

(b)

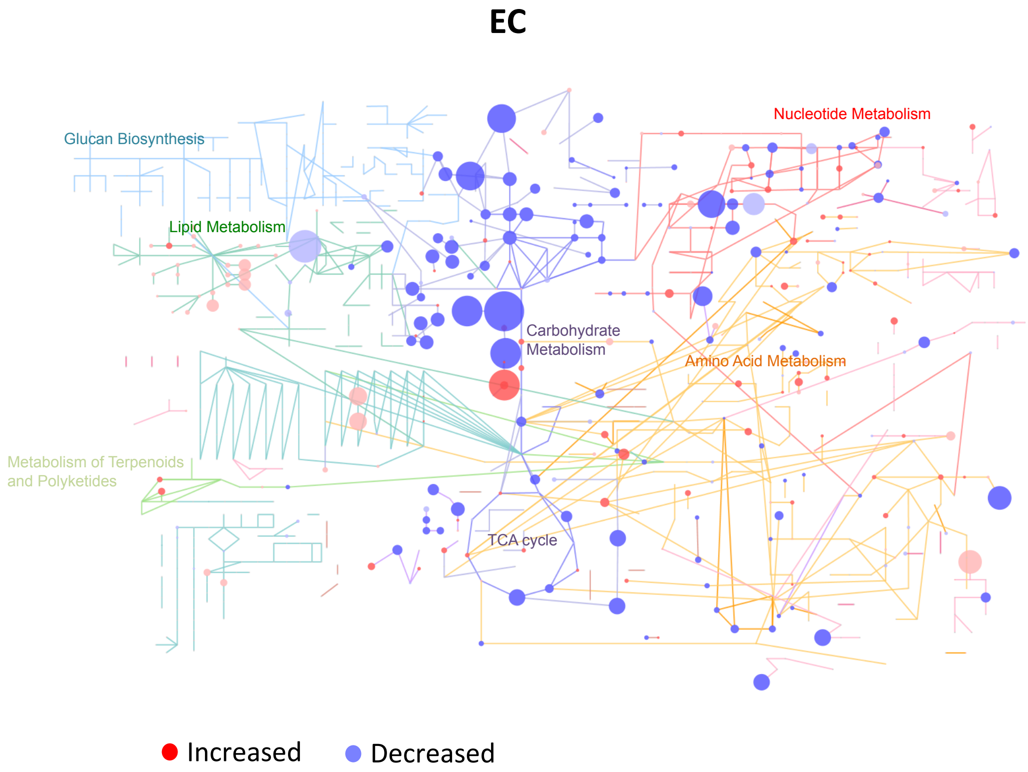

Fig. S4 Differential cellular modulation of glycogenolysis and glycolysis

(a)

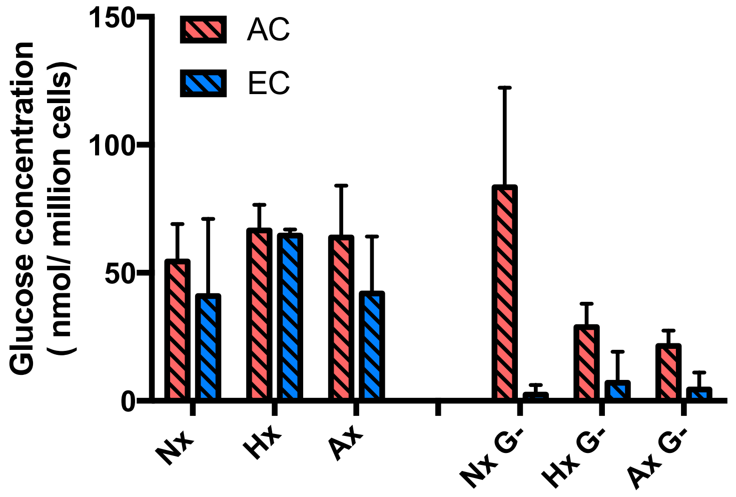

(b)

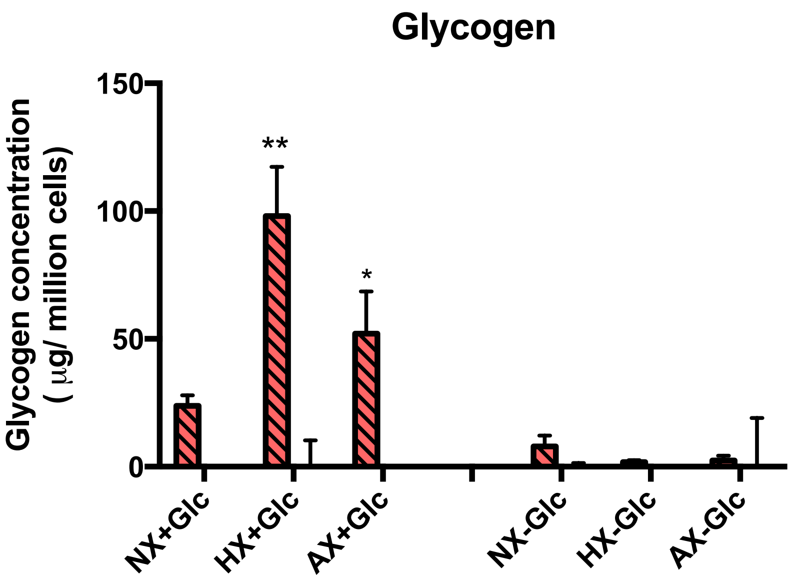

Fig. S5 Strong CR biosynthesis by AC but not EC

(a)

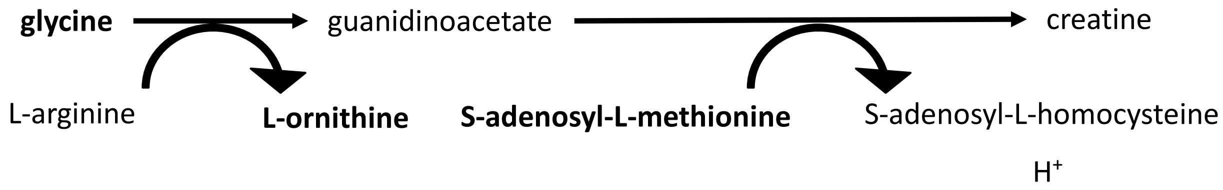

(b)

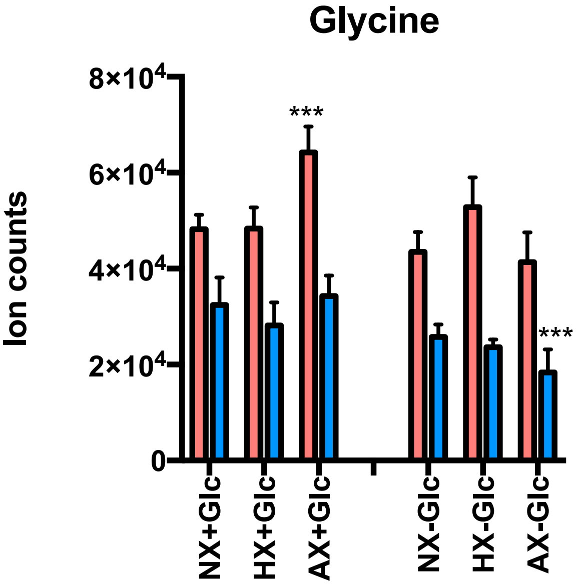

(c)

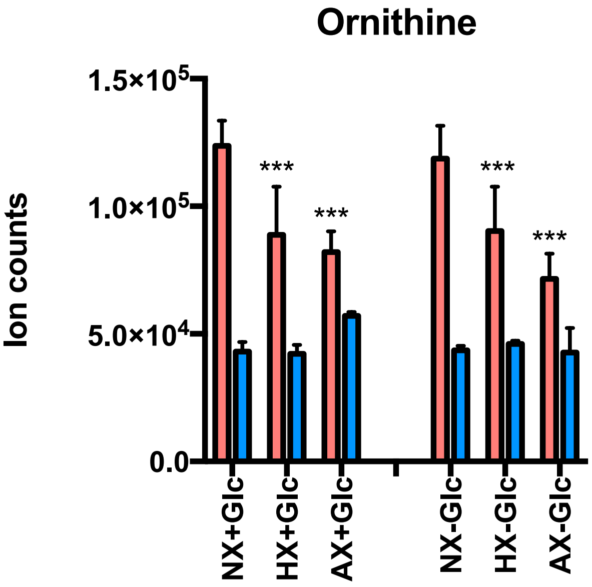

(d)

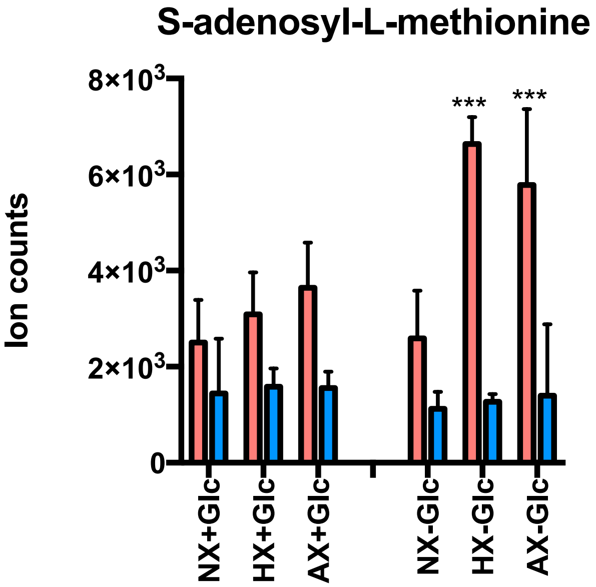

Supplement: Supplementary file 1 — Supplementary Figures. [file 41598_2020_64722_MOESM1_ESM.pdf]
